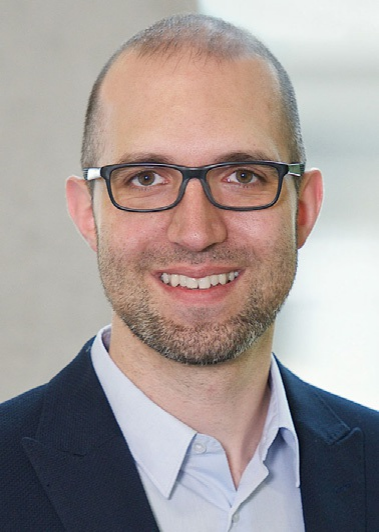# Inhibitors of Bacterial Swarming Behavior

**DOI:** 10.1002/chem.201901961

**Published:** 2019-10-24

**Authors:** Sina Rütschlin, Thomas Böttcher

**Affiliations:** ^1^ Department of Chemistry Konstanz Research, School Chemical Biology, Zukunftskolleg University of Konstanz 78457 Konstanz Germany

**Keywords:** flagella, interactions, motility, natural products, quorum sensing

## Abstract

Bacteria can migrate in groups of flagella‐driven cells over semisolid surfaces. This coordinated form of motility is called swarming behavior. Swarming is associated with enhanced virulence and antibiotic resistance of various human pathogens and may be considered as favorable adaptation to the diverse challenges that microbes face in rapidly changing environments. Consequently, the differentiation of motile swarmer cells is tightly regulated and involves multi‐layered signaling networks. Controlling swarming behavior is of major interest for the development of novel anti‐infective strategies. In addition, compounds that block swarming represent important tools for more detailed insights into the molecular mechanisms of the coordination of bacterial population behavior. Over the past decades, there has been major progress in the discovery of small‐molecule modulators and mechanisms that allow selective inhibition of swarming behavior. Herein, an overview of the achievements in the field and future directions and challenges will be presented.

## Graphical abstract

**Hard to stop**: Bacterial swarming motility is a form of coordinated population behavior which is important for pathogenicity and antibiotic resistance. Consequently, controlling swarming is of major interest for targeting bacterial pathogens and achieving better understanding of microbial motility. Herein, the progress of the last decades in discovery and development of small molecule modulators and mechanisms to inhibit swarming is presented.

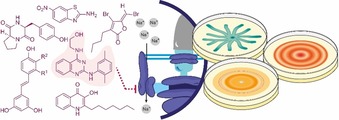

## 1 Introduction

Bacteria display numerous well‐regulated forms of population behavior to colonize ecological niches, cope with adverse conditions, and adapt to competitive or collaborative interactions with other species. Population behaviors range from the formation of sessile biofilms to various forms of cellular motility. One form of motility—the rapid movement of groups of flagellated cells across surfaces—is termed swarming.^1^ This behavior is driven by flagella in a thin‐liquid film on semi‐solid surfaces. Hereby, swarmer cells usually undergo cell differentiation leading to elongated snake‐ or rod‐shaped cells with multiple polar or peritrichous flagella.^2^ Other forms of bacterial motility include swimming behavior in three‐dimensional liquid space, pili‐driven twitching, or appendage‐independent forms of active gliding and passive sliding.^3^ Although mechanistically related, swimming involves movement of individual cells instead of the coordinated population behavior of groups of cells in swarming behavior.^1^ In some species, the types of flagella used for swarming motility are distinct from that used for swimming and adjustment of gel strength allows for the study of both forms of motility separately.3b Swarming represents maybe the most dynamic form of coordinated microbial behaviors that is controlled by multiple regulatory layers and consequently may be targeted in diverse ways by chemical modulators. These include global regulatory networks like for example master regulators, quorum sensing, two‐component systems, surface sensing, and protease activity and also sensing of environmental factors such as temperature and salt concentration.^4^ For most bacterial species, surface motility is facilitated by the production of surfactants, which also enable them to successfully colonize the host environments.3b

In this review article, we will primarily focus on the connection between swarming motility and small molecules and mechanisms allowing to control swarming.

So far, many questions such as why some bacteria swarm under certain conditions remain enigmatic. Following a lag phase, swarming colonies can reach expansion rates of about 5–36 mm h^−1^ and thereby cover an entire agar plate within several hours to a few days.^2^, 3b This rapid colonization of new area may be one of the ecological functions of swarming. Many human pathogens display swarming behavior and swarming also has biomedical relevance.^1^ Swarming was first described in 1885 for the urinary tract infective pathogen *Proteus mirabilis* and regarded as an undesired phenotype preventing the isolation of clinical strains from agar plates.^5^ Hence, the need for suppressing swarming behavior in cultures for diagnostic purposes was recognized early on. However, the relevance of swarming motility for the infection process itself was only discovered much later. Ever since, swarming motility has been associated with virulence of various important human pathogens such as *Pseudomonas aeruginosa*,^6^ *Escherichia coli*,^7^ *P. mirabilis*,^8^ *Vibrio cholerae*,^9^ *Salmonella typhimurium*,^10^ and *Clostridium septicum*.^11^ Many of these pathogens experience major shifts in the expression levels of virulence factors and other pathogenicity related traits correlating with formation of swarm cells. For example, swarming *P. mirabilis* displays increased virulence by hemolysin, ureolytic and proteolytic activities, and invasion behavior in comparison with nonmotile cells.^12^ The swarming phenotype also contributed to pathogenicity of *P. mirabilis* in infection models,^8^ and similarly in uropathogenic *E. coli* expression of flagella was found to be important for the colonization of the upper urinary tract.^7^ In *P. aeruginosa*, virulence is enhanced under swarming conditions by upregulation of gene expression of the type III secretion system as well as numerous virulence factors including extracellular proteases and the biosynthesis for siderophores and phenazines.^6^ Swarming behavior may further increase pathogenicity by facilitation of host attachment and colonization in various organisms ranging from humans to fungi and plants.^13^ In addition to increased virulence, swarming bacteria in many cases exhibit enhanced tolerance against different antibiotics compared with their planktonic counterparts.^6^, ^14^ High cell densities of swarming bacteria protected *S. typhimurium* even from several orders of magnitude higher concentrations of antibiotics than swimming cells which only move at low cell densities.^15^ Mixed species swarms also allow the transport of nonmotile bacterial species with mutual benefits, whereby a cargo species may contribute with antibiotic resistance mechanisms to the detoxification of the environment.^16^

Due to its impact on virulence and antibiotic tolerance, swarming motility is an important pathogenicity related trait. Inhibiting bacterial swarming behavior may thus have medical potential for treating or preventing infectious diseases. However, the molecular mechanisms involved in the regulation of swarming fundamentally differ from species to species and their detailed understanding is in many cases still incomplete.^17^ Surface motility requires the cells to overcome biophysical challenges such as surface wetting, friction, and surface tension.^18^ Also a wide range of environmental conditions, nutrients, and physical parameters influence swarming motility and diverse physical and chemical signals integrate into its regulation.^19^ Thus, swarming involves intertwined regulatory networks operating on metabolic, signal transduction, and gene‐expression level.^18^, ^19^ Consequently, strategies for swarming inhibition are diverse and involve a wide variety of different compound classes and modes of action. The literature on swarming modulation by small molecules is vast and dispersed across different research fields. Although many excellent reviews on bacterial motility and its biological regulation exist,^1^, ^17^, ^18^, ^19^ no informative and comprehensive overview on the chemistry of controlling swarming behavior has been reported so far. In this article we will review the current status and highlight new developments of swarming‐inhibitory compounds as well as provide mechanistic insights into their mode of action.

## 2 Swarming and Bacterial Signaling

One way bacteria regulate their swarming behavior is through chemical signals. Different types of signaling pathways exist, the most prominent of which are quorum‐sensing systems. Quorum sensing is a cell‐to‐cell signaling strategy inducing gene expression in dependence of bacterial population density. The corresponding small‐molecule signals are produced and accumulate during population growth. A receptor sensing these signals positively regulates transcription of various genes including genes for the biosynthesis of the signal itself—hence also called autoinducer. This synchronizes gene expression in a population‐density dependent manner and allows the coordinated production of virulence factors such as toxins, enzymes, or specific metabolites.^20^ Examples for signaling molecules are the widely distributed autoinducer 2 (AI‐2), the highly diverse class of *N*‐acyl‐homoserine lactones (*N*‐acyl‐HSLs or AHLs) in gram‐negative bacteria,^21^ as well as various autoinducing peptides (AIPs) in gram‐positive bacteria.^22^ Although in some species quorum‐sensing signals directly control swarmer cell differentiation, they regulate in others the production of biosurfactants that contribute to swarming motility by lowering surface tension. Examples of the latter are rhamnolipid of *P. aeruginosa* or surfactin of *Bacillus subtilis*.^23^ Given that quorum sensing has important impacts on swarming behavior, interference with its signaling can be applied to suppress swarming motility.

### 2.1 Inhibition of AI‐2 signaling

**Figure 1:**
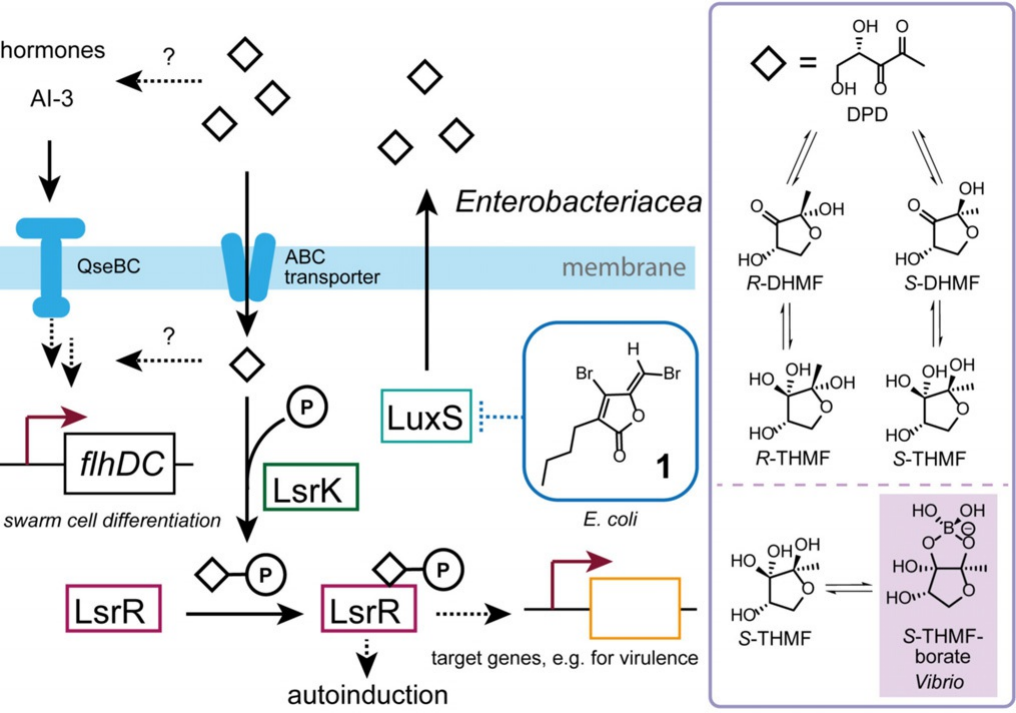
AI‐2 signaling and inhibition of signal synthesis through LuxS by the fimbrolide (**1**). The AI‐2 signal is *R*‐THMF in enterobacteria and the boric acid ester of *S*‐THMF for *Vibrio* species.

Although AI‐2 is the most common quorum‐sensing signal used by many different species and produced by gram‐negative as well as gram‐positive bacteria, only a few approaches have been reported in which AI‐2 signaling has been targeted for swarming inhibition. For *E. coli*, swarming‐cell differentiation has been shown to be regulated by the central FlhC_2_D_2_ master regulator the transcription of which is presumably activated by AI‐2 through the two‐component system QseBC (Figure 1). The FlhC_2_D_2_ regulator in turn activates the *fliA* gene which encodes a sigma factor specific for flagellar operons.4b In pathogenic *E. coli* strains, AI‐2 plays an important role for virulence and a nanoemulsion of 2.5 % limonene was found to interfere with AI‐2 quorum sensing of *E*. *coli* O157:H7 (EHEC). Hereby, both swimming and swarming motilities were repressed.^24^ The biosynthesis of the AI‐2 signal is carried out through cleavage of *S*‐ribosylhomocysteine by LuxS (Figure 1).^25^ For signal detection, AI‐2 is phosphorylated and derepresses transcription of target genes through binding to LsrR.^26^ Fimbrolides, a class of halogenated furanones, are important inhibitors of the LuxS signal synthase and thereby of quorum sensing by AI‐2.^27^ Fimbrolides have been initially discovered as natural products from the marine red alga *Delisea pulchra* and a great diversity of natural and synthetic derivatives has been investigated.^28^ A furanone (**1**) inhibited biofilm formation and swarming but not swimming motility in *E. coli* and strongly antagonized the quorum sensing by AI‐2.^29^ The same furanone also inhibited swarming of *B. subtilis*.^30^

### 2.2 Blocking AHL receptors

**Figure 2:**
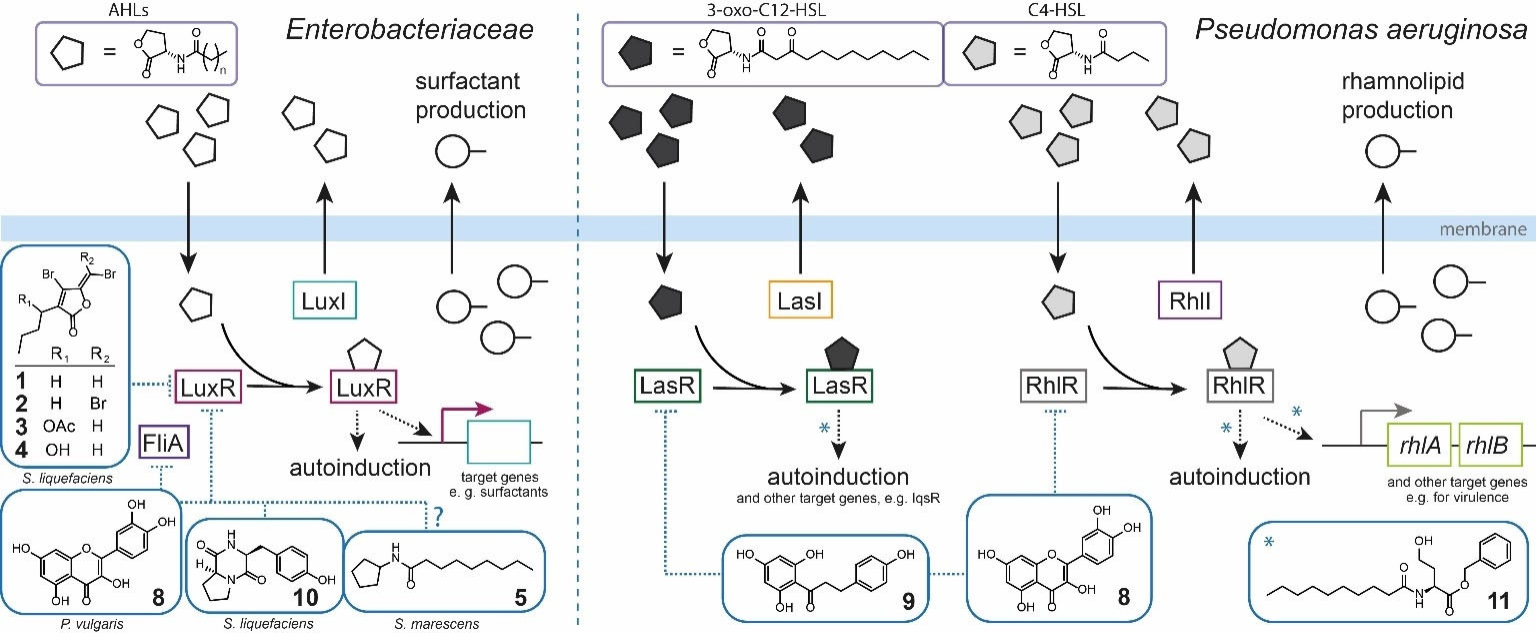
AHL‐based quorum sensing in enterobacteria (left) and *Pseudomonas aeruginosa* (right) and corresponding inhibitors that lead to inhibition of swarming motility.

Halogenated furanones have been additionally described to target the LuxE subunit of the luciferase complex of *Vibrio* and *N*‐acyl‐homoserine lactones (AHL)‐based quorum sensing through destabilization of homologues of the LuxR‐regulator.^27^, ^31^ AHLs are the largest class of quorum‐sensing signals in gram‐negative bacteria that are produced through N‐acylation of *S*‐adenosyl‐l‐methionine (SAM) and cyclization to γ‐lactones by homologues of the synthase LuxI (Figure 2, left). The signals are detected by binding to homologues of the transcription factor LuxR.^32^ In many species, AHLs have major impact on swarming regulation because they are regulators of, for example, the biosynthesis of the surfactant serrawettin through LuxR in *Serratia* spp. (Figure 2, left). Serrawettin promotes swarming motility by reduction of surface tension. Consequently, targeting AHL‐based quorum sensing has been of central interest for swarming inhibition. Two differently brominated furanones (**1**) and (**2**) of *D. pulchra* inhibited AHL‐dependent swarming motility of the enterobacterium *Serratia liquefaciens* which was restored in an AHL‐negative mutant by supplementation with *N*‐butanoyl‐l‐homoserine lactone (C4‐HSL).28b The mechanism of swarming inhibition involves the blockage of the biosynthesis of the surfactant serrawettin W2 as mentioned above through binding to LuxR.^33^ Surprisingly, only one of four brominated furanones isolated from *D. pulchra* inhibited swarming of the uropathogen *P. mirabilis*.^34^ All four furanones (**1**–**4**) inhibited swarming of different uncharacterized environmental strains of bacteria isolated from rock surfaces as well as from samples of *D. pulchra*.^35^

Targeting AHL receptors (LuxR homologues) has been maybe the most frequently employed strategy for interfering with AHL‐based quorum sensing. Especially AHL signal analogs that mimic the native AHLs are promising candidates for inhibitors. For example, AHL signaling can be inhibited by synthetic N‐acyl cyclopentylamides (Figure 2, left).^36^ A mutant strain of enterobacterium *Serratia marcescens* that was unable to produce AHLs was nonmotile in a swarming assay. Exogenous supply of *N*‐hexanoyl‐l‐homoserine lactone (C6‐HSL) restored the swarming phenotype and competition with 50 μm *N*‐nonanoyl cyclopentylamide (**5**) resulted in complete swarming inhibition.^37^

**Figure 3:**
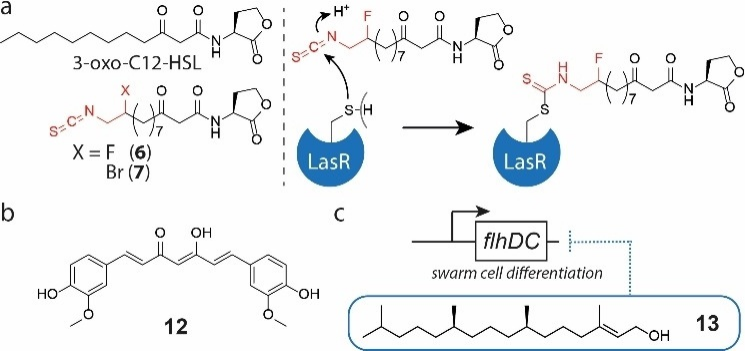
a) Covalent inhibition of LasR by the 3‐oxo‐C12‐HSL analogue ICT‐F (**6**) causing reduction in swarming motility in *P. aeruginosa*. Quorum sensing and swarming inhibitors b) curcumin and c) phytol which causes down‐regulation of *flhDC* expression.

Some species such as the human pathogen *P. aeruginosa* even comprise more than one AHL‐based quorum sensing system. In *P. aeruginosa*, the LuxI/LuxR homologues RhlI/RhlR and LasI/LasR utilize the signals *N*‐butanoyl‐l‐homoserine lactone (C4‐HSL) and *N‐(*3‐oxododecanoyl)‐l‐homoserine lactone (3‐oxo‐C12‐HSL), respectively (Figure 2, right). These AHL‐based quorum‐sensing systems are hierarchically interconnected by the master regulator LasR with further quorum‐sensing and two‐component systems to control virulence in *P. aeruginosa*.^38^ Recently discovered clinical isolates of *P. aeruginosa* from cystic fibrosis patients revealed an exceptional plasticity in the hierarchical regulation of quorum sensing whereby the RhlI/RhlR system could compensate the loss of functional LasR.^39^ The production of the swarming surfactant rhamnolipid which *Pseudomonas* requires to lower surface tension is RhlR regulated by transcription of the *rhl* genes. The Meijler group developed synthetic AHLs with an isothiocyanate (ITC) warhead mimicking 3‐oxo‐C12‐HSL of *P. aeruginosa*.^40^ These compounds and especially a β‐fluorinated derivative ICT‐F (**6**) covalently blocked the LasR receptor at Cys79 and inhibited swarming motility by 44 % at 150 μm and by 34 % at 20 μm and also reduced pyocyanin production (Figure 3 a). In contrast, the brominated ITC‐Br (**7**) did not bind covalently and was a LasR agonist that increased swarming motility up to 2.5‐fold at 20 μm of ITC‐Br in *P. aeruginosa* PA14.^40^

High‐throughput screening of a compound library against reporter strains revealed the plant‐produced flavonoids phloretin, chrysin, and naringenin as potent inhibitors of the LasR and RhlR quorum‐sensing receptors of *P. aeruginosa*.^41^ Additionally, also flavonoids like quercetin (**8**), baicalein, and pinocembrin exhibited inhibitory activity whereby the presence of a specific pattern of two hydroxyl‐groups on the flavonoid A‐ring appeared to be required for activity (Figure 2).

Flavonoids were found to be allosteric inhibitors of these quorum‐sensing receptors and prevented their binding as transcription factors to DNA. Two of the most active compounds, phloretin (**9**) and 7,8‐dihydroxyflavone were finally tested on quorum‐sensing‐controlled behaviors of *P. aeruginosa* and completely abrogated swarming at 100 μm.^41^ The flavonoid quercetin (**8**) considerably reduced swarming motility of *P. aeruginosa* and *Yersinia enterocolitica* at 132 μm.^42^ In *Proteus vulgaris*, 50 μm of quercetin (**8**) not only inhibited the production of *N*‐octanoyl‐l‐homoserine lactone (C8‐HSL) by 81 % and caused an almost equal reduction in swarming area, but also supposedly interfered with swarming by binding to the sigma factor FliA which regulates flagellar operons (Figure 2, left).^43^ A virtual docking‐approach against the AHL receptor LasR identified salicylic acid and chlorzoxazone as potential quorum‐sensing inhibitors of *P. aeruginosa* which was confirmed biochemically through LasR and additionally RhlR and resulted in inhibition of swarming of *S. liquefaciens* in the millimolar range.^44^ Also complex natural‐product mixtures and extracts have been found to exhibit quorum‐sensing inhibiting activities affecting swarming behavior. For example, propolis—bee glue—antagonized AHL‐based quorum‐sensing signaling in RhlR‐ and LasR‐dependent reporter strains and reduced swarming activity of *P. aeruginosa*.^45^ Some signals may even lead to crosstalk between different quorum‐sensing systems. An example are diketopiperazines (DKPs), cyclic dipeptides involved in trans‐kingdom interactions of bacteria with eukaryotes^46^ and inter‐species signaling between gram‐negative and gram‐positive bacteria.^47^ DKPs such as cyclo(ΔAla‐l‐Val), cyclo(L‐Pro‐l‐Tyr) (**10**), and cyclo(l‐Phe‐l‐Pro) were isolated from culture supernatants of various gram‐negative bacteria including Pseudomonads, *P. mirabilis*, *Citrobacter freundii*, and *Enterobacter agglomerans* and recombinant LuxR‐based AHL biosensor assay revealed that they compete with the site of AHL binding and thereby antagonize quorum sensing. Cyclo(l‐Pro‐l‐Tyr) (**10**) reduced swarming of wild type *S. liquefaciens* as well as of a Δ*swrI* mutant for which swarming motility depends on external supply of *N*‐butanoyl‐l‐homoserine lactone (C4‐HSL) (Figure 2, left).^48^

In many cases, however, the cellular targets of quorum‐sensing inhibitors or their compound classes have not yet been clearly identified. Hereby, phenotypic or transcriptional analyses have often tentatively pointed to interference with AHL‐based quorum sensing as likely mechanism of swarming inhibition. An AHL‐derived *N*‐decanoyl‐l‐homoserine benzyl ester (**11**) for example inhibited swarm expansion and dendritic swarming pattern between 50 and 100 μm and reduced expression of both *las* and *rhl* genes as well as production of virulence factors including rhamnolipids (Figure 2, right).^49^ At 136 μm and higher concentrations, curcumin (**12**) inhibited swarming motility of *E. coli*, *P. aeruginosa* PAO1, *P. mirabilis*, and *S. marcescens* and interfered with AHL‐based quorum sensing in a violacein assay (Figure 3 b).^50^ At high concentrations of around 1.5 mm, caffeine inhibited AHL production in *P. aeruginosa* and reduced swarming motility^51^ and zingerone inhibited swarming, swimming, and twitching motility at 5 mm and also decreased the production of AHLs.^52^ Many further natural products and synthetic compounds have been postulated to inhibit quorum sensing of *P. aeruginosa* at relatively high concentrations through LasR whereby swarming motility, but not growth, was inhibited. Examples are, *trans*‐anethole with a reduction of swarming motility by 64 % at 6 mm 53 or pyridoxal lactohydrazone with a reduction of swarming motility by about 35 % at 32 μm and ≈70 % at 126 μm.^54^

The non‐methylated version of the pyrrolidin alkaloid (*R*)‐norbgugaine superficially resembles 3‐oxo‐C12‐HSL and inhibited swarming motility and production of virulence factors of *P. aeruginosa*.^55^ The anti‐inflammatory drugs diclofenac and also ketoprofen were shown to inhibit swarming motility of *P. aeruginosa* at 5 mm concentration without any growth inhibition. Reduced production of virulence factors as well as activity in an AHL‐quorum‐sensing inhibition screen suggested that these compounds inhibited swarming through the quorum‐sensing circuits with the molecular targets yet to be identified.^56^ A diazaborine‐based copolymer with quorum‐sensing inhibitory activity in a violacein assay showed swarming inhibition by about 50 % against *P. aeruginosa* PAO1 at a concentration of 100 μg mL^−1^, whereas the MIC (minimal inhibitory concentration) was determined to be 10 times higher.^57^ At relatively high concentrations of 10–12 mm, the food additives diallyl disulfide (DADS) and methyl 2‐methyl‐3‐furyl disulfide (MMFDS) inhibited C6‐HSL production of the enterobacterium *Hafnia alvei*, reduced expression levels of *luxI* and *luxR* and inhibited swarming by more than 70 %.^58^

In *S. marcescens*, production of its red pigment prodigiosin is under control of AHL‐based quorum sensing. Methanolic extracts of the benthic brown alga *Padina gymnospora* inhibited production of this pigment and activity guided fractionation led to α‐bisabolol as active compound. Furthermore, α‐bisabolol inhibited extracellular protease, biofilm formation and swarming motility at and above 450 μm suggesting interference with AHL‐based quorum sensing as mechanism. Swarming was abolished completely at 1.8 mm without inhibiting growth.^59^ At much lower concentrations between 17 and 34 μm, phytol (**13**) reduced virulence factor production of *S. marcescens* and strongly inhibited swarming motility (Figure 3 c).^60^ The activity of phytol was presumably mediated through quorum‐sensing inhibition because it resulted in transcriptional down‐regulation of many quorum‐sensing‐controlled genes including the swarming differentiation master‐regulator genes *flhC* and *flhD*. Finally, treatment of rats with phytol in an acute pyelonephritis model even ameliorated the infection with *S. marcescens*.^60^

### 2.3 Interspecies activity of alkyl quinolone signals

**Scheme 1:**
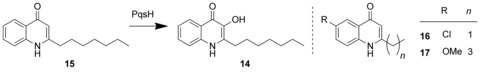
The native metabolites HHQ (**15**) and PQS (**14**) and synthetic derivatives with swarming inhibitory activity.

*P. aeruginosa* comprises a multi‐layered network of intertwined quorum‐sensing systems regulating its virulence and population behaviors like swarming. In addition to the two AHL‐based quorum‐sensing systems introduced previously, *P. aeruginosa* also utilizes an alkyl quinolone‐based system as well as the more recently discovered integrated quorum‐sensing (IQS) system.^38^ The alkyl quinolone‐based systems signal through congeners of the Pseudomonas Quinolone Signal (PQS, (**14**)) and its biosynthetic precursor HHQ (**15**) and the receptor PqsR (also known as MvfR) and possibly many further interaction partners (Scheme 1).^61^ In *P. aeruginosa*, PQS as well as C4‐HSL are known to regulate the transcription of *rhl*R genes, thus modulating rhamnolipid production. In addition, HHQ and PQS have been implicated in interspecies and even interkingdom interactions.^62^ For example, PQS at 50 μm inhibited swarming of *Pseudomonas putida* and reduced biofilm formation by interference with signaling and iron‐uptake.^63^ HHQ and PQS also repressed swarming and flagella‐independent forms of motility in other gram‐negative and gram‐positive bacteria.62b Although the mechanism of motility reduction by HHQ and PQS remained obscure in this study it was presumably unrelated to their role as quorum sensing signals since homologs of the PQS signaling system are restricted to only a few species of *Pseudomonas* and *Burkholderia*.^64^ *P. aeruginosa* shares a common environment with *Bacillus atrophaeus* in soil and PQS completely abrogated swarming of *B. atrophaeus* at 10 μm, whereas HHQ at the same concentration only led to minor reduction of swarming.62b, 65 Development of synthetic HHQ derivatives with substitutions at the anthranilate‐derived ring of the quinolone core and variations of the alkyl chain resulted in several potent compounds with enhanced anti‐swarming activity. Two of them (**16** and **17**) even completely abrogated swarming motility of *B. atrophaeus* (Scheme 1).^65^

### 2.4 Enzymatic quenching of the signal

In addition to disrupting AHL signaling through inhibition of its production or blocking of the signal receptor, also enzymatic degradation of the signal itself is leading to quorum quenching and altered swarming behavior.^66^ This can be accomplished by lactonases which hydrolyze the γ‐lactone ring of AHLs. An example is provided by the mammalian paraoxonase enzyme family that degraded and thus quenched AHL‐based quorum sensing of *P. aeruginosa* whereby swarming was significantly reduced already at concentrations of 3 μg mL^−1^ of human serum paraoxonase 1.^67^ Another lactonase Ahl‐1 from *Bacillus weihenstephanensis* isolate‐P65 at 0.5 mg mL^−1^ also inhibited AHL accumulation and reduced virulence‐factor production and swarming of *P. aeruginosa*.^68^

Screening of a metagenomic library revealed HqiA as novel AHL lactonase family enzyme that quenched AHL signals and the *hiqA* gene introduced in the swarming plant pathogen *Pectobacterium carotovorum* reduced its motility and production of virulence‐related maceration enzymes.^69^

Given that HHQ and PQS inhibit swarming of several bacterial species, enzymatic quenching of these molecules by other bacterial species may affect motility in interspecies interactions. For example, the dioxygenase Hod from *Arthrobacter nitroguajacolicus* and the enzyme Aqd from *Mycobacterium abscessus* have been described as PQS‐degrading enzymes.^70^ So far, however, effects of these enzymes on HHQ‐ and PQS‐mediated swarming inhibition still remain to be demonstrated.

### 2.5 Other signaling systems

**Figure 4:**
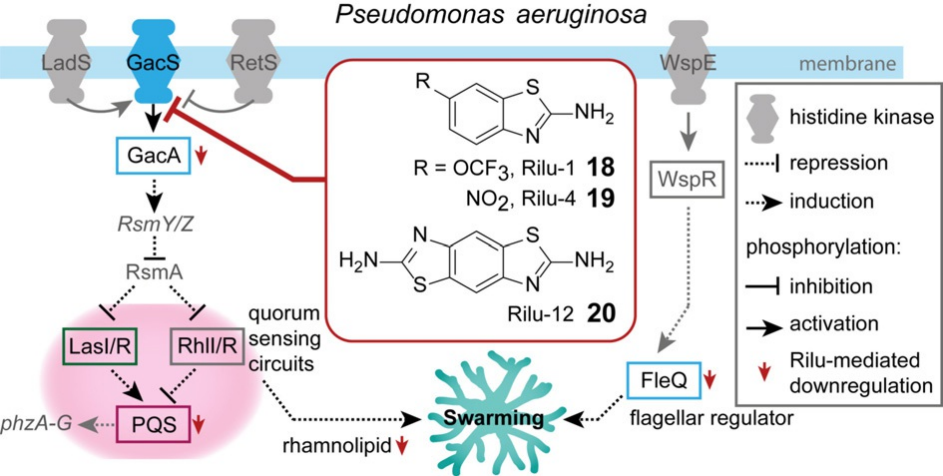
Inhibition of swarming by histidine kinase inhibitors targeting the two‐component system GacSA.

In addition to its multiple quorum‐sensing systems, *P. aeruginosa* also comprises a large diversity of distinct two‐component systems regulating virulence.^71^ Each of them is composed of a histidine kinase (HK) sensing external stimuli and a response‐regulator protein that alters gene expression upon phosphorylation by the kinase. The many two‐component systems for *Pseudomonas* have been shown to be intricately involved in swarming regulation for example through the action of the response regulator GacA, which is activated by the HK GacS. GacA is connected to swarming through the RhlI/RhlR system through several regulatory steps. Benzothiazole‐based histidine kinase inhibitors (Rilu‐1 (**18**), Rilu‐4 (**19**), and Rilu‐12 (**20**)) reduced PQS signaling, decreased rhamnolipid production and drastically impaired swarming motility at 200 μm (Figure 4). Gene‐expression analysis suggested that these benzothiazoles inhibited the sensory kinase GacS whereby the transcription of the response regulator *gacA* and also the flagellar regulator *fleQ* was decreased.^72^ In some cases also chemoattractants may be important for swarming motility. This was demonstrated for *P. mirabilis* on minimal medium, in which swarming depended on the amino acid l‐glutamine as signal lead to swarmer‐cell differentiation and up‐regulation of the expression of flagellin (*fliC*) and hemolysin (*hpmA*). The glutamine‐analogue γ‐glutamyl hydroxamate interfered with this signaling and inhibited swarming at 10 mm.^73^

Consequently, chemical signaling and the modulation of its activity by small molecules is a promising strategy for controlling swarming and other population behaviors in different species. The diversity of signaling pathways even within a single species such as *P. aeruginosa* and the manifold interactions of microbial signals across species give rise to a large and yet only partially explored chemical space for specific and selective inhibitors of swarming behavior.

## 3 Sub‐Inhibitory Concentrations of Antibiotics

**Scheme 2:**
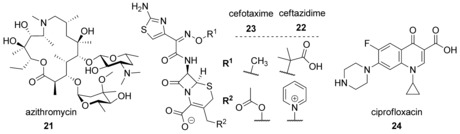
Antibiotics inhibiting swarming at sublethal concentrations.

Antibiotics are highly important drugs against pathogenic bacteria that contribute immensely to human health. Many antibiotics are naturally produced by soil microbes and it has been proposed that some antibiotics may even have roles in the ecosystem beyond inhibiting growth of competitors.^74^ These antibiotics are regarded to serve at sub‐lethal concentrations, that is, below MIC as cell–cell communication signals and regulate transcription of certain genes, including that of important virulence factors.^75^ Accordingly, some antibiotics control at low concentrations microbial behavior and also affect swarming motility. The macrolide azithromycin, for example, showed swarming‐inhibitory effects against *P. aeruginosa* and *P. mirabilis* in various studies. Hereby, the best inhibition with azithromycin (**21**) was at a concentration of about 21 μm (1/16 MIC) with more than 80 % inhibition of swarming of *P. aeruginosa* PAO1 (Scheme 2).^76^ In another study, 11 μm azithromycin inhibited the swarming of 15 clinical isolates of *P. aeruginosa* from 18 to 73 %, whereas swarming of all clinical isolates of *P. mirabilis* was already completely inhibited at 5 μm.^77^ Swarming inhibition by azithromycin correlated with suppressed expression of flagellin in *P. aeruginosa* and *P. mirabilis*.^78^ Azithromycin also reduced expression of *lasI*/*lasR* and *rhlI*/*rhlR* in *P. aeruginosa* and inhibited AHL production.^76^, ^79^ Some macrolide antibiotics like erythromycin and clarithromycin also inhibited swarming and flagellin expression,^78^ whereas for example the macrolide rokitamycin had no effect on the expression of flagellin and consequently did not inhibit swarming.^78^

Also, β‐lactam antibiotics as inhibitors of cell‐wall biosynthesis affect virulence and population behavior at concentrations below the MIC. For example, the third‐generation cephalosporin ceftazidime (**22**) inhibited virulence of *P. aeruginosa* PAO1 and PAF97 and reduced swarming motility by around 80 % at 0.9 and 3.7 μm, respectively (Scheme 2).^80^ The antibiotics cefotaxime (**23**), ciprofloxacin (**24**), chloramphenicol, and trimethoprim completely blocked swarming of the gram‐negative pathogen *Salmonella enterica* (ser. Typhimurium) at sub‐growth inhibitory concentrations of 3.5, 0.02, 6, and 3 μm, respectively. In contrast, amikacin, colistin, kanamycin, and tetracycline did not inhibit swarming of *S. enterica* (ser. Typhimurium). While cefotaxime (**23**), ciprofloxacin (**24**) and trimethoprim inhibited polar‐chemoreceptor array assembly of *S. enterica* (ser. Typhimurium) that is essential for swarming, chloramphenicol inhibited swarming by a decrease in flagellation (Scheme 2).^81^

Many further antibiotic classes have been linked to regulatory effects on bacterial behavior at sublethal concentrations.75c For example, also the aminoglycoside gentamicin, like azithromycin (**21**), reduced *lasI*/*lasR* and *rhlI*/*rhlR* expression and AHL production in *P. aeruginosa* and considerably impaired swarming motility at approximately 0.2 μm (1/16 MIC) by over 70 %.^76^ At 1/4 of the MIC, gentamicin (MIC≈0.06–0.2 mm) and amikacin (MIC=1.7–3.4 μm) resulted in 30–60 % swarming inhibition of various clinical isolates of *P. mirabilis*.^82^ The gyrase inhibitors nalidixic acid and novobiocin completely inhibited swarming of *E*. *coli* at 20 and 200 μm, respectively.^83^ In the lower micromolar range, also sulfonamides such as sulfamethazin blocked swarming of the majority of 250 strains of *P. mirabilis* and *P. vulgaris* tested.^84^ Doxycycline was reported to inhibit swarming of *P. aeruginosa* PAO1 in the lower micromolar range with more than 60 % inhibition at 4.5 μm likely through targeting of quorum sensing.^85^

In addition, different antibiotic peptides inhibited swarming at sublethal concentration. For example, the naturally occurring pseudopeptide antibiotic actinonin at below MIC concentrations between 0.05 and 0.5 μm reduced swarming motility of *S. enterica* (ser. Typhimurium) and *Vibrio vulnificus*.^86^ A small cationic peptide (KRFRIRVRV‐NH_2_) with weak antibiotic activity considerably inhibited (by >70 %) at sub‐MIC concentration of 4 μm the swarming motilities of *P. aeruginosa* PAO1 and PA14 and *Burkholderia cenocepacia*. Hereby, the transcription of several flagellar genes and *rhlB* for rhamnolipid production was downregulated.^87^ A series of cationic antimicrobial peptides with repeating tryptophan–arginine motif was tested against the swarming of *E. coli*. In this study, the hexapeptide (RW)_3_‐NH_2_ showed the strongest swarming inhibition with almost complete blockage of swarming at a concentration of 25 μm.^88^ Cationic peptides are known to exhibit their antimicrobial activity by targeting cell membranes^89^ and may thus also disrupt flagellar integrity.^88^

The inhibition of swarming motility at low concentrations appears to be a common theme for many but not all antibiotics. In some cases, like amikacin, swarming inhibition even seems to be species specific.^81^, ^82^ Although the mechanisms by which antibiotics in low concentrations inhibit swarming behavior are so far not conclusively understood, targeting of quorum sensing as well as direct interference with the regulation of flagellar gene expression or flagellar integrity are likely central concepts. Antibiotics also may lead to long‐term regulatory changes in bacterial cells which have been pre‐exposed for extended time to sublethal concentrations of antibiotics. For example, pretreatment of *E. coli* with approximately 1 μm (1/2 MIC) gentamicin through downregulation of succinate dehydrogenase (*sdh*) genes inhibited swarming but not swimming motility in a fumarate‐dependent manner.^90^ Fumarate metabolism also was found to be important for swarming motility of *P. mirabilis*.^91^ Also a continuous low‐dose pre‐exposure of *P. aeruginosa* to erythromycin (2 μm) and clarithromycin (1 μm) for 2–18 months led to approximately 70 % reduction of swarming motility and attenuated virulence although it did not affect the MIC value.^92^ Whether these effects are caused or facilitated by genetic mutations or entirely rely on regulatory changes that prevail for several generations after antibiotic exposure has so far not been investigated.

## 4 Secondary Plant Metabolites

**Scheme 3:**
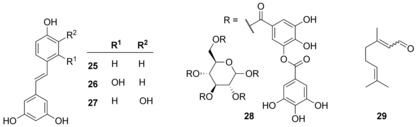
Examples of secondary plant metabolites with swarming‐inhibitory activity.

Plants produce an enormous diversity of secondary metabolites and great deal of research has focused on natural products and their effects on bacterial population behaviors including swarming motility. For example, different plant extracts inhibited swarming of *E. coli* O157:H7 (EHEC) whereby extracts of the sedge grass *Carex dimorpholepis* containing high concentrations of the phytoalexine *trans*‐resveratrol were the most potent. Swarming of EHEC was inhibited by 44 μm *trans*‐resveratrol (**25**) which correlated with transcriptional repression of the motility genes *flhD*, *fimA*, *fimH*, and *motB* (Scheme 3).^93^ At 263 μm *trans*‐resveratrol completely inhibited swarming of *P. mirabilis* and significantly reduced swarming already at 66 μm. A mutant of the gene *rsbA* restored swarming of *P. mirabilis* in presence of *trans*‐resveratrol with preserved flagellin production and elongated‐cell phenotype, suggesting that the regulatory protein RsbA mediates inhibition of swarmer cell differentiation by *trans*‐resveratrol.^94^ Resveramax, a formulation of *trans*‐resveratrol further inhibited swarming of *P. aeruginosa* and global effects on quorum‐sensing‐related phenotypes were observed.^95^ In another study, also *trans*‐oxyresveratrol (**26**) and *trans*‐piceatannol (**27**) almost completely abolished swarming of *P. aeruginosa* between 100 and 200 μm without inhibiting growth (Scheme 3). Transcription analysis revealed downregulation of the *las* and *rhl* quorum‐sensing regulatory circuits.^96^ The structurally related chlorogenic acid only slightly inhibited swarming of *P. aeruginosa* but also exhibited global effects on quorum‐sensing‐controlled virulence factors.^97^ The compound (*Z*,*Z*)‐5‐(trideca‐4′,7′‐dienyl)‐resorcinol that was isolated from the plant *Lithrea molleoides* significantly inhibited swarming motility of *P. mirabilis* at 28 μm and completely abolished swarming at 433 μm.^98^ Furthermore, many similar plant‐derived phenolic compounds including caffeic acid, cinnamic acid, ferulic acid, and vanillic acid have been reported to inhibit swarming of *P. aeruginosa* at 4 mm.^99^

Also, tannins such as proanthocyanidins are important phenolic compounds produced by many plant species. Cranberry proanthocyanidin extracts and pomegranate extracts containing the related punicalagin completely abolished swarming of *P. aeruginosa* at 100 μg mL^−1^ without inhibiting growth. Both extracts did not affect swimming motility. Addition of rhamnolipid partially restored swarming, suggesting that the mechanism involved repression of biosurfactant production.^100^ Cranberry products also transiently impaired swarming of urinary tract infective *P. mirabilis*.^101^ More defined tannins such as pure epigallocatechin gallate and tannic acid (**28**) blocked swarming of *P. aeruginosa* down to approximately 20 and 3 μm, respectively (Scheme 3).^102^

In contrast, methyl gallate, which corresponds to a structural motif of tannic acid only exhibited low swarming inhibitory activity against *P. aeruginosa* in the range of several hundred micromolar.^103^ Neutralized tannic acid at 12 mm (0.02 % (w/v)) also inhibited the swarming of all 27 strains of *P. mirabilis* tested.^104^

Many further plant metabolite classes inhibit swarming. Examples are terpenes of which citronellol poorly inhibited swarming of *P. mirabilis* at 1.9 mm 105 and the related citral (**29**) which considerably inhibited swarming motility of the food‐borne pathogen *Cronobacter sakazakii* already at 113 μm and repressed various virulence genes.^106^ At millimolar concentrations also the red pigment brazilin from the wood of the *Caesalpinia* family,^107^ cinnamaldehyde,^108^ and 2‐phenethylamine^109^ inhibited swarming motility of different species. A 10′(*Z*),13′(*E*)‐heptadecadienylhydroquinone (HQ17‐2) isolated from the lacquer tree inhibited swarming motility of *P. mirabilis* between 36 and 145 μm through the two‐component system RcsB which controls the *flhDC* genes encoding the flagellar master regulator FlhD_2_C_2_.^110^

With exception of tannins, plant metabolites exhibited comparably low activity on swarming bacteria. The mechanisms hereby may be as diverse as the compound classes and range from inhibition of surfactant production to regulatory effects on flagellar gene expression.

## 5 Off‐Target Effects of Synthetic Compounds

**Scheme 4:**
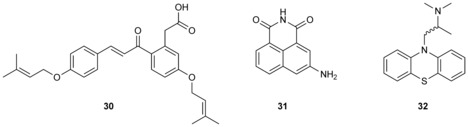
Drugs with off‐target effects that inhibit swarming behavior.

Off‐target activities of drugs, pesticides, and other xenobiotics have in some cases also led to inhibition of swarming behavior. The gastrointestinal drug solfacone (**30**) for example, which is also present in herbs used in traditional Chinese medicine, significantly inhibited *Heliobacter pylori* swarming at a concentration of 22 μm without any growth inhibition (Scheme 4).^111^ 3‐Amino 1,8‐naphthalimide (**31**), an analogue of virstatin, a compound targeting the cholera toxin regulator ToxT, was highly effective against swarming of *V. cholerae* at a concentration of about 12 μm without any effect on the growth of the bacteria. This effect could be attributed to an inhibition of chemotaxis, but the secondary target was not further identified.^112^ The drug ambroxol, commonly used in asthma and chronic bronchitis, completely inhibited swarming motility of *P. mirabilis* at high concentrations of 2.4 mm.^113^

Furthermore, the effects of a range of psychotropic drugs was tested against another *Proteus* and *Proteus*‐related strains. Of these compounds, the antihistamine promethazine (**32**) exhibited the best inhibition effects against *P. vulgaris* at 150 μm, which was several times lower than the MIC value (Scheme 4).^114^ Swarming inhibition could be antagonized by K^+^ and Na^+^ ions, suggesting that interference of promethazine with ion homeostasis would adversely impact flagellar motility.^114^

Different psychotropic drugs were also tested against *P. vulgaris*, *P. mirabilis*, and *Morganella morganii*, whereby the antidepressant sertraline inhibited all strain's swarming motility at about 100 μm independent of its MIC which was 2–16 times higher.^115^ Swarming of *P. mirabilis* and *P. vulgaris* was efficiently blocked by the synthetic compound *p*‐nitrophenyl glycerol, which completely abolished swarming at 0.1 and 0.2 mm for more than 24 h and depending on culture conditions even for >80 h, whereas growth was only affected above 0.5 mm. However, swarming cells exposed to *p*‐nitrophenyl glycerol seemed to have developed resistance and resumed swarming motility sooner than unexposed cells.^116^ *p*‐Nitrophenyl glycerol has been used in clinical laboratories to block swarming for bacterial isolation and also other studies reported complete swarming inhibition for *Proteus* between 0.2 and 0.7 mm as well as downregulation of virulence factors.^117^ Many different mycotoxins, fungicides, insecticides, and herbicides affected in the upper micro‐ to millimolar range the swarming motilities of *P. mirabilis* and *Azospirillum brasilense*.^118^ The chromogenic β‐galactosidase substrate 5‐bromo‐4‐chloro‐3‐indolyl‐β‐d‐galactopyranoside (X‐Gal) reduced or inhibited swarming of different *Vibrio* species, including *V. cholerae*, *Vibrio mimicus*, *V. vulnificus*, *Vibrio alginolyticus*, and *Vibrio parahaemolyticus* at 235 μm without affecting viability but facilitated swarming motility of *P. mirabilis* and *S. marcescens*.^119^ Although the mode of swarming inhibition of most of these compounds remains unexplored, their pharmacophore properties, as well as their relatively potent activities, suggest specific interference with cellular processes required for bacterial motility that warrant further investigation.

## 6 Fatty Acids

Swarming motility is dependent on many factors like for example the population density and the concentration of sodium ions. Furthermore, the surface wetness of the solid medium is fundamentally important and a challenge for standardizing swarming assays.3b, 120 In many species, swarming relies on the control of surface tension and wetness by the secretion of surfactants. Modulating the secretion of surfactants is a mechanism that can stall swarming colonies and this mechanism has been reported for the swarming inhibitory activity of various fatty acids. For example, the branched‐chain fatty acid 12‐methyltetradecanoic acid selectively and completely inhibited swarming motility of *P. aeruginosa* PAO1 at a concentration of 41 μm without affecting growth.^121^ The effect could be assigned to a general repression of secreted surfactants which also included surface‐active precursors of rhamnolipids.^122^ Surfactant production of *P. aeruginosa* has been also blocked by the supplementation of swarming plates with halogenated alkanoic acids. These compounds directly inhibit the biosynthesis of polyhydroxyalkanoic acid (PHA) and rhamnolipids through inhibition of the enzymes PhaG and RhlA, respectively, and thus block surfactant‐mediated swarming motility. 2‐Bromohexanoic acid was hereby found to be the most potent congener inhibiting swarming at 2 mm.^123^

The swarming inhibition by fatty acids can be further attributed to the modulation of regulatory systems associated with swarming motility. The saturated fatty acids dodecanoic and tetradecanoic acid completely blocked swarming motility of a clinically isolated *S. marcescens* strain at 0.01 % (wt/vol) supplemented to swarming plates. The effect, which turned out to be dose‐dependent, resulted mainly in a delay in the swarming lag time. Swarming inhibition was hereby associated with the saturated fatty acid‐regulated two‐component regulatory system RssAB.^124^ Another non‐QS‐regulated mechanism was found to be responsible for the swarming inhibition of *S. marcescens* by petroselinic acid (*cis*‐6‐octadecenoic acid) at 0.7 mm which was associated with a 0.8‐fold downregulation of the swarming motility master regulator genes *flhDC*.^125^

## 7 Amphiphilic Compounds

**Figure 5:**
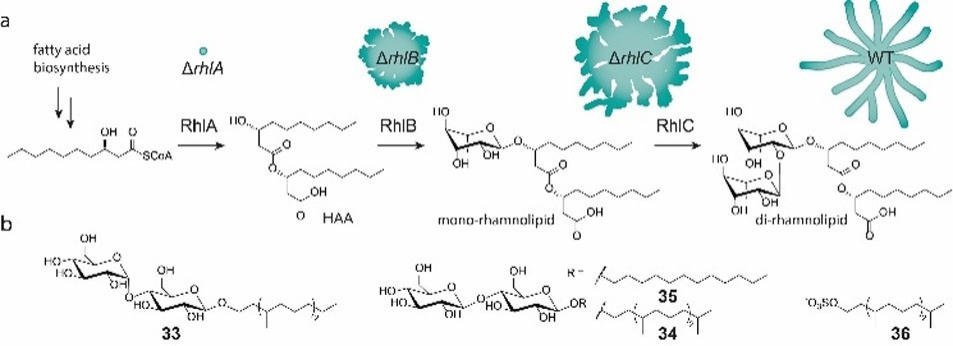
Surfactants controlling swarming behavior of *P. aeruginosa*. a) Biosynthesis of rhamnolipids and swarming pattern of genetic knockout strains of the indicated biosynthesis genes. b) Synthetic surfactants modulating or inhibiting swarming motility of *P. aeruginosa*.

In addition to fatty acids, also other surface‐active substances are known to inhibit swarming. The swarming inhibiting effect against *P. mirabilis* in the case of homologous sodium alkylsulfates increased with chain length from hexyl‐ (20–30 mm) to tetradecyl sulfate (0.1–0.5 mm) without impaired growth.^126^ At 0.5 mm, sodium tetradecyl sulfate completely inhibited swarming of *P. mirabilis* and impaired swarming already at 0.1 mm supposedly either by inhibition of formation of flagella or lysis of existing flagella.117c The effect of 58 chemical substances including detergents and surfactants was tested against *Bacillus* swarming.^127^ Sodium dodecyl sulfate and bile salts such as sodium taurocholate and sodium desoxycholate strongly inhibited or completely blocked swarming of different strains of *B. subtilis*, *Bacillus alvei*, *Bacillus coagulans*, and *Bacillus circulans* in the lower millimolar range, whereas polysorbates (Tween 20–80) even promoted swarming.^127^ Bile salts also inhibited swarming of enterobacteria such as *P. mirabilis*.^128^ Rhamnolipids of *P. aeruginosa* are a class of native surfactants with dual roles in reducing surface tension and modulating tendril formation. Although a *rhlA* mutant deficient in biosynthesis of all rhamnolipids as well as their β‐d‐(β‐d‐hydroxyalkanoyloxy)alkanoic acid (HAA) precursor is unable to swarm, the *rhlB* and *rhlC* mutants exhibit altered, irregular tendril patterns (Figure 5 a).^129^ Purified rhamnolipids even can inhibit swarming of wild‐type *P. aeruginosa*, demonstrating their important roles in spatial modulation of motility in swarming colonies.^129^ A library of synthetic farnesyl‐modified disaccharides mimicking rhamnolipids of *P. aeruginosa* PAO1 was explored for effects on swarming motility and quorum sensing.^130^ Many of these compounds promoted swarming at low concentrations and inhibited swarming at higher concentrations. While the farnesylated disaccharides SFβM (**33**) and SFβC (**34**) completely inhibited swarming of wild type *P. aeruginosa* PAO1 already at 20 and 25 μm, respectively, the closely related compound DβC (**35**) with a dodecyl chain rescued a *rhlA* mutant at 20 μm and did not inhibit swarming of wild type PAO1 up to 85 μm (Figure 5 b). This indicates that also the lipid component has major impact for controlling motility. A sulfate functionalized saturated farnesol (**36**) even inhibited swarming completely between 5 and 10 μm (Figure 5 b). It was proposed that different saccharide or lipid‐binding receptors in the outer membrane may have been responsible for these activities.^130^ Similar to some fatty acids discussed before, these rhamnolipid mimetics may thus act on regulatory level.

The endosymbiont *Burkholderia gladioli* of the beetle *Lagria villosa* produces the antibiotic lipocyclopeptide icosalide which is an interesting example for the intraspecies regulation of swarming by amphiphilic compounds. Although linear lipopeptides of *B. gladioli* promoted swarming, icosalide inhibited swarming motility indicating that their interplay may regulate host colonization and free‐living lifestyles.^131^

## 8 Interference with Flagellar Motor Assembly and Function

**Figure 6:**
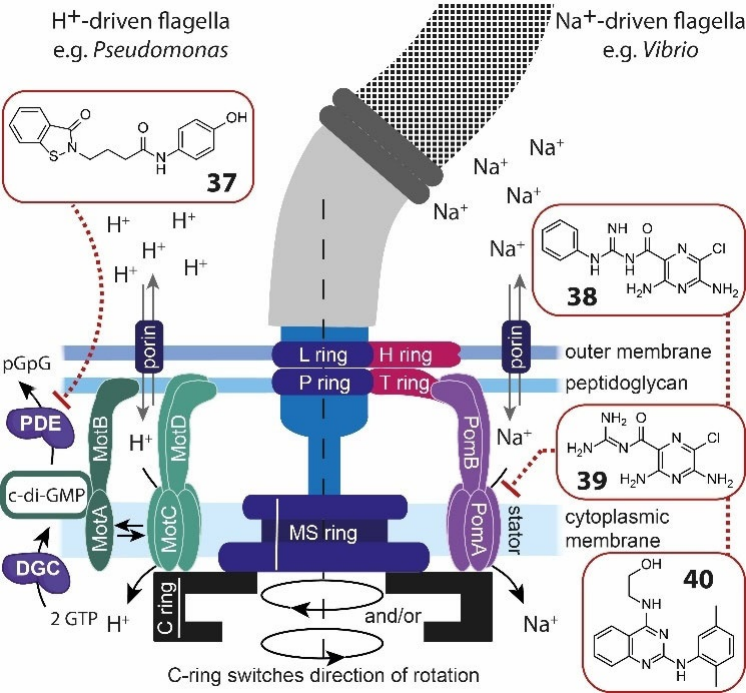
Flagellar motor assembly of H^+^‐ and Na^+^‐driven flagella and compounds interfering with motor function causing swarming inhibition.

Each bacterial flagellum consists of a long helical protein filament which connects through a hook to the basal body in the cell envelope. Rotation of the motor complex in the membrane is powered by the transport of protons or sodium ions across the membrane. The rotor is surrounded by a ring of membrane‐anchored stator complexes that comprise the corresponding ion channels and their interactions with the rotor generate the torque for the rotation of the flagellum (Figure 6). Most bacterial species possess multiple stator systems which can engage in highly dynamic rotor–stator interactions tuning the flagellar motor.^132^ The incorporation and exchange of stators in the motor complex depends on diverse environmental factors like the level of viscous drag or sodium‐ion concentration but is also regulated by the intracellular second messenger cyclic diguanylate (c‐di‐GMP).^133^ In *P. aeruginosa*, motility is mediated by one rotor with two sets of stators, MotAB and MotCD. Although MotCD is required for swarming, the MotAB stator represses swarming motility. Under high c‐di‐GMP concentrations stator selection is in favor of MotAB and thereby c‐di‐GMP inhibits swarming.^134^ Also in other species elevated c‐di‐GMP levels lead to inhibition of motility.^135^ Intracellular c‐di‐GMP levels are controlled by multiple diguanylate cyclases (DGCs) which produce c‐di‐GMP from two molecules of GTP and phosphodiesterases (PDEs) that hydrolyze c‐di‐GMP (Figure 6). Different DGCs and PDEs may hereby control c‐di‐GMP on local and global scale in the cell and integrate diverse signals and stimuli.^136^ In a positive feedback regulation, disengaged MotCD stators further stimulate DGC activity, thereby block motility and support biofilm formation.^137^ Inhibitors of DGCs and PDEs can be designed to modulate c‐di‐GMP levels. Zheng et al. reported a benzoisothiazolinone derivative (**37**) which was found by in silico screening against the structure of an *E*. *coli* PDE.^138^ This compound inhibited selectively c‐di‐GMP hydrolysis of the locally acting PDE RocR of *P. aeruginosa* with a *K*_i_ of 83 μm, but did not inhibit three other PDEs of *P. aeruginosa* whereby global cellular c‐di‐GMP levels remained unaffected (Figure 6). Inhibition of RocR at 100 μm completely suppressed swarming but did not increase biofilm production.^138^

Another strategy to interfere with swarming motility involves direct blocking of the corresponding flagellar motor. Phenamil (**38**) and amiloride (**39**) are inhibitors of Na^+^‐driven motors and have been used to dissect motor functions in different bacterial models such as *Vibrio* and *Bacillus* (Figure 6).^139^ Both compounds are pyrazine derivatives that block the Na^+^‐channels of the stator complexes and thus prevent generating torque for flagellar rotation.139a High‐throughput screening for swarming inhibitors of *V. cholerae* resulted in a 2,4‐diamino quinazoline (**40**) and derivatives which inhibited swarming with IC_50_ values in the single‐digit micromolar range (Figure 6). These compounds blocked Na^+^‐driven flagellar motors of different *Vibrio* species but had no effect on the proton‐driven flagellar motors of E. *coli* and the lateral flagella of *V. parahaemolyticus*.^140^

## 9 Phages Modulating Motility

Flagellar function can also be impaired by certain bacteriophages. Phages can infect bacteria either by the direct exploitation of their host resulting in phage replication and host‐cell lysis (lytic) or by integrating into the bacterial genome and being replicated along with bacterial‐cell division (lysogenic). Although a lysogenic infection as such typically has no effect on bacterial motility, *P. aeruginosa* PA14 lysogenized with the bacteriophage DMS3 was unable to swarm and form biofilms. This inhibition depended on CRISPRs as well as five of the six *cas* genes of the host that, when deleted, restored the swarming and biofilm‐forming phenotype.^141^ Flagellotrophic phages physically attach to their host's flagella and have been found to infect only motile cells.^142^ Yet, effects on motility of the host bacteria have been rarely reported. The flagellotrophic phage χ_7_ has a broad host range of various species of bacteria. By contact with *P. mirabilis*, this phage rendered its host immediately nonmotile and swarming of more than 85 % of clinical *Proteus* isolates was inhibited without killing of the bacteria.^143^ Thus, specific bacteriophages are able to impair swarming possibly on regulatory level or by direct physical interactions. So far, the detailed mechanisms of how phages interfere on regulatory level or physically disable flagellar motility remain obscure.

## 10 Interspecies Competition and the Microbiota

**Figure 7:**
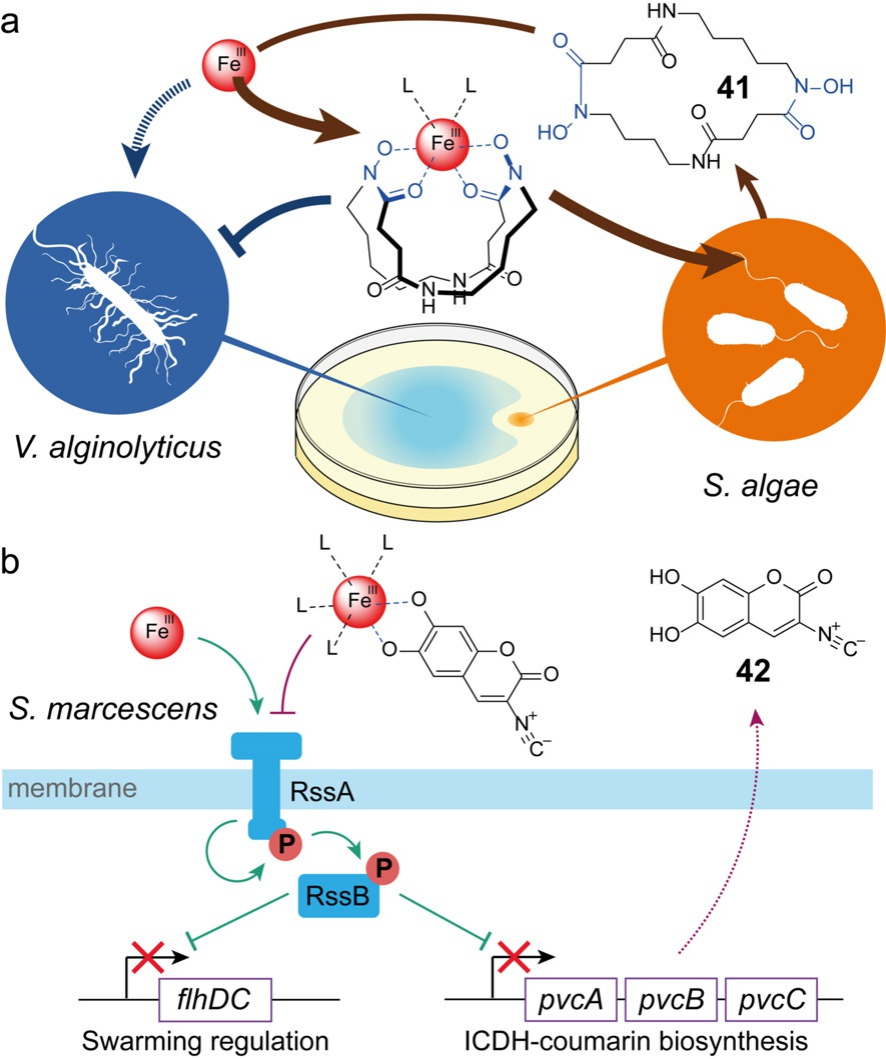
Swarming and bioavailability of ferric iron. a) Avaroferrin produced by *Shewanella algae* blocks iron‐dependent swarming motility of *Vibrio alginolyticus*. b) Chelation of ferric iron by the ICDH‐coumarin **42** switches off RssAB two‐component system signaling and thereby triggers swarming.

Competitive chemical interactions of bacteria play an important role in multi‐species communities in many different environments. Thus, many species may have evolved small molecules to modulate population behaviors of their competitors to their own benefit. This includes interference with swarming motility. For example, the marine bacterium *Marinobacter litoralis* inhibited swarming of *P. aeruginosa* by its lipopolysaccharide (LPS) whereas LPS from other species did not affect motility.^144^ In another study, the methanol extracts of 72 *Actinomycetes* isolated from marine invertebrates were screened for activity against *P. aeruginosa*. Extracts of two strains inhibited at 0.1 mg mL^−1^ swarming of *P. aeruginosa* by 90 and 85 %, the major active component of which was cinnamic acid.^145^ In addition to small molecules, proteins also may contribute to competitive interactions. This was observed for the soil bacterium and human pathogen *Burkholderia pseudomallei* that secreted a protein factor to inhibit swarming of *Burkholderia thailandensis* by damaging or processing of its flagella.^146^ Also the competition for resources can influence bacterial motility. Essential trace elements such as ferric iron are highly embattled in the microbial world and bacteria compete for ferric iron by deploying siderophores as high‐affinity iron chelators. Availability of ferric iron also controls swarming behavior of *V. parahaemolyticus* and *V. alginolyticus*.^147^ Although in *V. parahaemolyticus* iron limitation is essential for swarmer‐cell differentiation,147a *V. alginolyticus* requires bioavailability of ferric iron for swarming. To sequester ferric iron from the environment, *V. alginolyticus* encodes many different iron‐siderophore receptors in its genome that allow the bacterium to engage in piracy of siderophores produced by other species. A strain of *Shewanella algae* which was co‐isolated with *V. alginolyticus* from the same seaweed sample evaded this siderophore piracy by producing avaroferrin (**41**) (Figure 7 a)—a chimera of the homodimeric macrocyclic hydroxamate siderophores putrebactin and bisucaberin.^148^ In a disc‐diffusion assay on agar, avaroferrin (50 nmol) led to the formation of a zone with inhibited swarming motility of *V. alginolyticus* whereas the homodimeric siderophores were considerably less active.^149^ Other siderophores were inactive (>500 nmol), whereas deferasirox, an artificially optimized iron chelator for which no receptor in *V. alginolyticus* is available was a potent swarming inhibitor like avaroferrin. These results suggested that evasion of siderophore piracy by the chimeric siderophore of *S. algae* limited ferric iron uptake and thereby stalled swarming of *V. alginolyticus*.^149^ This mechanism was confirmed by exploiting the promiscuity of the central NRPS‐independent siderophore (NIS) synthetases giving access to non‐natural ring‐size engineered siderophores, which inhibited swarming of *V. alginolyticus* with potency comparable to avaroferrin.^150^ In contrast, *S. marcescens* swarms only under limitation of ferric iron which is sensed by a two‐component system through the endogenously produced iron chelator 2‐isocyano‐6,7‐dihydroxycoumarin (**42**) (ICDH‐Coumarin) (Figure 7 b).^151^

Competitive interactions within the microbiota of higher organisms may shape health and disease of their eukaryotic host.^152^ Particularly interesting hereby are the abilities of commensal and probiotic microbes to protect their hosts from pathogens. For example, the swarming pathogen *S. marcescens* causes the white‐pox disease in corals by colonizing and penetrating the coral's mucus layer. Commensal bacteria were isolated from the coral *Acropora palmata* and investigated for their ability to compete with *S. marcescens*. In co‐culturing experiments, strains of *Photobacterium damselae*, *Photobacterium leiognathi* and *Vibrio harveyi* induced a clear swarming inhibition zone of *S. marcescens*, the active compounds, however, have not yet been identified.^153^ Interactions between microbial species can also be found within the human microbiota. For instance, culture supernatants of probiotic *Lactobacillus acidophilus* and *Lactobacillus plantarum* were active against the swarming motility of *S. marcescens* and completely inhibited swarming at 2 % (v/v).^154^ Lactic acid produced by a probiotic *Pediococcus* strain inhibited at sub‐MIC concentrations the production of short‐chain AHLs as well as swarming and swimming motility of clinical isolates of *P. aeruginosa*. However, there was no evidence that short‐chain AHL inhibition was causal for inhibiting motility.^155^ Various microorganisms share the ability to oxidize bicyclic aromatic compounds like naphthalene and indole. The oxidation products 1‐naphtol as well as different hydroxyindoles completely blocked swarming motility of *P. aeruginosa* at 50 μm. The activity was found not to be related to changes in c‐di‐GMP levels or rhamnolipid production and was restricted to inhibition of swarming but not swimming motility.^156^ Human pathogens also may compete with each other, which has been for example reported by the ability of hemolytic *E. coli* but not *P. aeruginosa* or *Acinetobacter baumannii* to completely block swarming of *P. mirabilis*.^157^ In addition to microbe–microbe interactions, swarming motility can be influenced by metabolites of the human host. This has been demonstrated for urea which inhibited at around 0.5–1 % swarming of the urinary tract‐infective human pathogen *P. mirabilis*.^158^ Human urine contains approximately 1.5 % of urea (250 mm) and may thus represent a first line of defense against colonization by this pathogen.^159^

## 11 Summary and Outlook

An enormous diversity of approaches has been reported that allows to control the swarming behavior of different bacterial species. Swarming inhibitors cover the wide range from simple fatty acids over structurally complex secondary plant metabolites to enzymes intercepting bacterial signals and phages that block flagellar motility. Equally diverse are the mechanisms involved in inhibition of swarming and inhibitors have already contributed largely to our understanding of flagellar function and the different levels of regulatory control. Many swarming inhibitors have been demonstrated or proposed to interfere on regulatory levels. However, mechanism‐based inhibitors targeting signal production with a covalent mode of action such as halogenated furanones represent only a marginal group. The majority of compounds seems to interfere with signal receptors and transcription factors controlling gene expression. Although indirect effects through quorum sensing cannot always be ruled out, at least several compounds appear to directly interfere with flagellar gene expression or the flagellar master regulator. In addition to the regulation of flagellar genes, also inhibition of surfactant production is in some cases responsible for blocking motility. Other compounds even may directly impair flagellar integrity or interfere with motor function.

Microbe–microbe interactions may still hold great potential for the discovery of novel swarming inhibitors. Although potent effects of extracts have been already reported, the active compounds have largely remained uncharacterized. Especially interactions within the human microbiota between commensal and pathogenic microbes may lead to swarming inhibitors that could help to dissect the roles of swarming for health and disease of the human host. Understanding the corresponding chemistry and mechanisms could also allow to exploit microbial competition for the customized control of microbial populations and interactions. Currently, in vivo application presents a major challenge which may require new generations of swarming inhibitors. So far potent anti‐swarming activity has been rare. Particularly effective were antibiotics at sublethal concentrations and selected surfactants that inhibited swarming in the lower micromolar range. However, many swarming inhibitors were of rather low efficacy and only partially reduced motility or only blocked swarming at substantially high concentrations of several hundred micromolar or even millimolar. Although we tried to focus on compounds that genuinely block swarming and do not simply reduce motility as a side effect of growth inhibition, it is generally challenging to distinguish both effects. Especially when compounds are cytotoxic at higher concentrations, growth inhibition must be carefully evaluated. Also swarming inhibition of a compound was frequently overcome at longer incubation times. This limited number of highly active inhibitors may be explained by the altered physiological state of swarmer cells and cell‐density effects which also cause increased antibiotic tolerance of swarming bacteria. These challenges will have to be overcome for the development of customized high‐efficacy swarming inhibitors to allow in vivo applications in animal models and finally also in humans. Blocking swarming motility may exhibit future potential for use in combination therapies by decreasing virulence and host colonization while increasing antibiotic susceptibility of bacterial pathogens.

## Conflict of interest

The authors declare no conflict of interest.

## Biographical Information

*Sina Rütschlin studied Chemistry from 2010 till 2015 at the University of Konstanz. She joined the group of Thomas Böttcher in 2015 as a fellow of the fast‐track PhD programme of the Konstanz Research School Chemical Biology and obtained her PhD in May 2019. She currently continues her work as postdoctoral researcher. Her research is focused on the modulation of bacterial swarming behavior and biosynthesis of cyclic hydroxamate siderophores*.

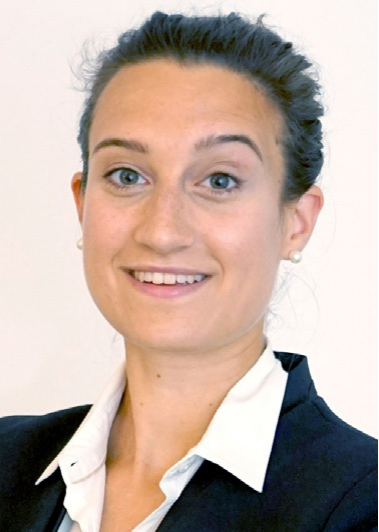

## Biographical Information

*Thomas Böttcher studied Chemistry and Biochemistry at the LMU in Munich and obtained his PhD in 2009 in the group of Stephan Sieber. Based on his research, he co‐founded the drug development start‐up company AVIRU GmbH. Supported by a Leopoldina research fellowship he conducted postdoctoral work with Jon Clardy at Harvard Medical School in Boston. Since 2014 he is head of an independent Emmy Noether research group at the University of Konstanz*.